# Electrical Capacitors Based on Silicone Oil and Iron Oxide Microfibers: Effects of the Magnetic Field on the Electrical Susceptance and Conductance

**DOI:** 10.3390/mi15080953

**Published:** 2024-07-25

**Authors:** Ioan Bica, Eugen Mircea Anitas, Gabriela Eugenia Iacobescu

**Affiliations:** 1 Department of Physics, West University of Timisoara, V. Parvan Avenue 4, 300223 Timisoara, Romania; ioan.bica@e-uvt.ro; 2 Department of Physics, Craiova University, A. I. Cuza Street 13, 200585 Craiova, Romania; gabriela.iacobescu@edu.ucv.ro; 3 Joint Institute for Nuclear Research, 141980 Dubna, Russia; 4 Horia Hulubei, National Institute of Physics and Nuclear Engineering, 077125 Magurele, Romania

**Keywords:** electrical capacitors, silicone oil, iron oxide microfibers, magnetic field, electrical conductance, electrical susceptance

## Abstract

This paper presents the fabrication and characterization of plane capacitors utilizing magnetodielectric materials composed of magnetizable microfibers dispersed within a silicone oil matrix. The microfibers, with a mean diameter of about 0.94 μm, comprise hematite (α-Fe_2_O_3_), maghemite (γ-Fe_2_O_3_), and magnetite (Fe_3_O_4_). This study investigates the electrical behavior of these capacitors under the influence of an external magnetic field superimposed on a medium-frequency alternating electric field, across four distinct volume concentrations of microfibers. Electrical capacitance and resistance measurements were conducted every second over a 60-s interval, revealing significant dependencies on both the quantity of magnetizable phase and the applied magnetic flux density. Furthermore, the temporal stability of the capacitors’ characteristics is demonstrated. The obtained data are analyzed to determine the electrical conductance and susceptance of the capacitors, elucidating their sensitivity to variations in microfiber concentration and magnetic field strength. To provide theoretical insight into the observed phenomena, a model based on dipolar approximations is proposed. This model effectively explains the underlying physical mechanisms governing the electrical properties of the capacitors. These findings offer valuable insights into the design and optimization of magnetodielectric-based capacitors for diverse applications in microelectronics and sensor technologies.

## 1. Introduction

Advancements in capacitor technology are pivotal for the development of modern electronic devices and systems [1,2,3,4,5]. Electrical capacitors are essential components in a wide range of applications, from energy storage systems [6,7,8] to advanced sensors [9,10,11] and transducers [12,13,14]. The performance and functionality of capacitors are significantly influenced by the properties of the dielectric materials used, which has led to ongoing research and innovation in this field [15,16]. They form components of an electrical circuit that consist of two or more electrodes with a dielectric material placed between them.

Various types of electrical capacitors are known, each defined by the dielectric material used. For instance, some capacitors utilize dielectric materials such as oil-impregnated paper [17], mineral oil [18], graphene oxide films [19], edge-free three-dimensional graphene [20], α-Fe_2_O_3_, multilayer ceramic capacitors [3], or percolative polymer composites [21]. Another category includes electrolytic capacitors, where both the electrodes and the dielectric material are produced using nanotechnology processes [22,23]. These capacitors are specifically designed for storing electrical energy [24]. Additionally, there are capacitors based on silicon oil (SO) and magnetizable nano-microparticles. In these capacitors, the equivalent electrical components can be adjusted magnetically [25,26,27,28,29].

Andrei et al. [25] manufactured capacitors using suspensions based on SO, iron microparticles, and stearic acid with varying mass ratios. These capacitors are characterized by an increase in electrical conductance with the increase in the ratio of stearic acid to the magnetizable phase in a magnetic field. Conversely, the duration for establishing electrical conduction decreases slightly with the increasing intensity of the applied magnetic field. The capacitors detailed in Refs. [26,27] are based on commercially available cotton fabrics impregnated with liquid suspensions that include carbonyl iron microparticles and varying ratios of honey and turmeric powder. When studied in an alternating electric field with frequencies ranging from 25 Hz to 1 MHz, superimposed on a static magnetic field, the equivalent electrical capacitance and resistance of the capacitors are measured. These properties are coarsely adjusted by the ratios of honey to turmeric powder and the values of magnetic flux density, while fine adjustments are achieved through the frequency of the alternating electric field. The capacitors described by Bica et al. [28] are constructed from medical-grade cotton gauze impregnated with liquid composites containing multifloral honey, carbonyl iron microparticles, and varying amounts of turmeric powder. In these capacitors, electrical conductance is coarsely adjusted by the ratio of honey to turmeric quantities and finely tuned by the intensity of the electric field. Iacobescu et al. [29] produced capacitors using cotton fabric impregnated with a magnetic liquid based on mineral oil and magnetite nanoparticles. By maintaining a constant quantity of magnetic liquid, it was observed that the electrical conductance of the composite can be coarsely adjusted by applying compressive stress and finely tuned by the values of the magnetic flux density.

Following this research direction, the present study describes the manufacturing method of capacitors based on SO and microfibers containing α-Fe_2_O_3_, γ-Fe_2_O_3_, and Fe_3_O_4_. The study investigates the electrical behavior of these capacitors under the influence of an external magnetic field superimposed on an alternating electric field, across four distinct volume concentrations of microfibers. Electrical capacitance and resistance measurements are conducted every second over a 60-s interval, revealing significant dependencies on both the quantity of magnetizable phase and the applied magnetic flux density. The obtained data are analyzed to demonstrate that in a medium-frequency electric field, both electrical conductance and susceptance can be coarsely adjusted by varying the ratio of SO to microfibers and finely tuned by the magnetic flux density. Compared to the capacitors produced in [25,26,27,28,29], this study shows that the presence of semiconductor iron oxides in the microfibers alters the behavior of electrical conductance when a magnetic field is applied. A theoretical model based on dipolar approximations is proposed to explain the underlying physical mechanisms governing the electrical properties of the capacitors.

By providing valuable insights into the design and optimization of magnetodielectric-based capacitors, our findings can influence the development of advanced microelectronic devices and sensor technologies. Improved capacitors with adjustable electrical properties have the potential to enhance the performance and efficiency of energy storage systems, leading to more reliable and scalable renewable energy solutions. Additionally, the ability to fine-tune the electrical characteristics of capacitors through magnetic fields can lead to innovations in electronic circuits, allowing for more adaptable and multifunctional electronic components. This research contributes to the advancement of composites based on iron microfibers and SO, paving the way for new applications and improvements in existing technologies.

The remainder of this paper is organized as follows: In Section 2, we detail the materials and methods used in the preparation of the magnetodielectric materials and the fabrication of the electrical capacitors. Section 3 presents the theoretical model based on dipolar approximations, explaining the physical mechanisms governing the electrical properties of the capacitors. In Section 4, we report the experimental results, including the variation in electrical capacitance and resistance with time under different magnetic flux densities. We also discuss the implications of these results for the design and optimization of PECs. Section 5 offers a comprehensive discussion of the findings, comparing our results with previous studies and highlighting the potential applications of magnetodielectric-based capacitors in microelectronics and sensor technologies. Finally, Section 6 concludes the paper, summarizing the main contributions and suggesting directions for future research.

## 2. Materials and Methods

### 2.1. Preparation of Magnetodielectric Materials

**Figure A1:**
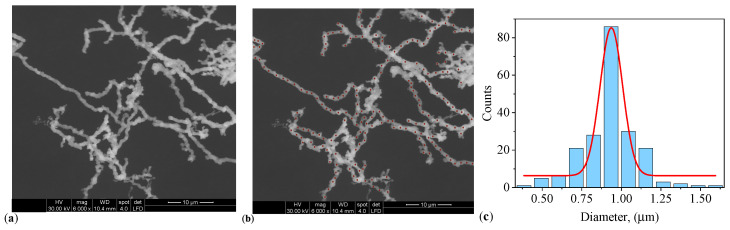
(**a**) SEM image of microfibers. (**b**) SEM image of microfibers with points (in red) where their thickness was measured. (**c**) Histogram of the thickness (diameter) of the microfibers (light blue columns) together with a Gaussian fit (red continuous line).

The materials used for the preparation of the magnetodielectric materials are as follows:

1. SO, from Siliconi Commerciale SpA (Italia), with a mass density of $\rho_{\mathrm{SO}}=0.97$ g/cm^3^ and dynamic viscosity $\eta_{\mathrm{SO}}=97$ mPa·s at T=295 K.
2. The iron oxide microfibers (mFe) are obtained in microwave microplasma, following a procedure described in Ref. [30]. They contain oxides of the type α-${\mathrm{Fe}}_{2}O_{3}$ (12% by mass), γ-${\mathrm{Fe}}_{2}O_{3}$ (62% by mass), and ${\mathrm{Fe}}_{3}O_{4}$ (26% by mass). The mass density of the microfibers is $\rho_{\mathrm{mFe}}=2.875\;{g/\mathrm{cm}}^{3}$ at T=295K and they exhibit a specific saturation magnetization, $\sigma_{\mathrm{mFe}}=22.7$ A·m^2^/kg, at a magnetic field intensity, H=477kA/m. A structural analysis of these microfibers based on SEM is presented in Appendix A. The average diameter of the nano-microparticles in the microfibers is $d_{m}=0.94\;\mu m\;\pm \;0.01\;\mu m$ (see Figure A1).

The magnetodielectric materials consist of mixtures of mFe and SO at four different concentrations, in the form of composite liquids (CL), as listed in Table 1. Each mixture $CL_{i}$ (i=1,2,3,4) is mechanically homogenized for a duration of about 300 s at a temperature T≈410 K. The homogenization of the samples continues until the mixtures are brought to ambient temperature (T≈300 K).

In the study of the magnetic properties of composite materials, the relationship used to determine the specific saturation magnetization $\sigma_{s_{\mathrm{CL}}}$ is $\mu_{0}\sigma_{s_{\mathrm{CL}}}=\mu_{0}\Phi_{\mathrm{mFe}}\sigma_{\mathrm{mFe}}$ [31], where $\mu_{0}$ is the vacuum magnetic permeability, $\Phi_{mFe}$ is the volume fraction of the mFe microfibers, and $\sigma_{\mathrm{mFe}}$ is the specific saturation magnetization. For $\sigma_{\mathrm{mFe}}=22.7$ A·m^2^/kg and the $\Phi_{\mathrm{mFe}}$ values from Table 1, when introduced into the specified relationship above, the $\sigma_{s_{\mathrm{CL}}}$ values (see Table 1) of the specific saturation magnetization of the liquids CL are obtained. One can see from Table 1 that increasing the amount of mFe microfibers results in increased values of $\sigma_{s_{\mathrm{CL}}}$.

### 2.2. Manufacturing Plane Electrical Capacitors

**Figure 1:**
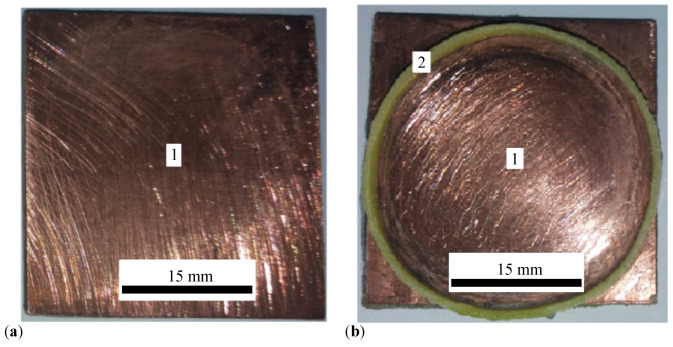
(**a**) PCB. 1—copper foil. (**b**) Cylinder. 1—copper foil, 2—dielectric ring.

The materials used for manufacturing the plane electrical capacitors (PECs) are the liquids CL_*i*_ (i=1,2,3,4) and a textolite plate, single-sided with copper foil. The printed circuit board (PCB), type LMM 100x210E1, is purchased from Electronic Light (Bucharest, Romania). The actual board is made from FR4-type epoxy resin, reinforced with fiberglass. On one side of the actual board, an electrolytic copper foil, with a thickness of 35 μm, is deposited (Figure 1a, pos. 1).

**Figure 2:**
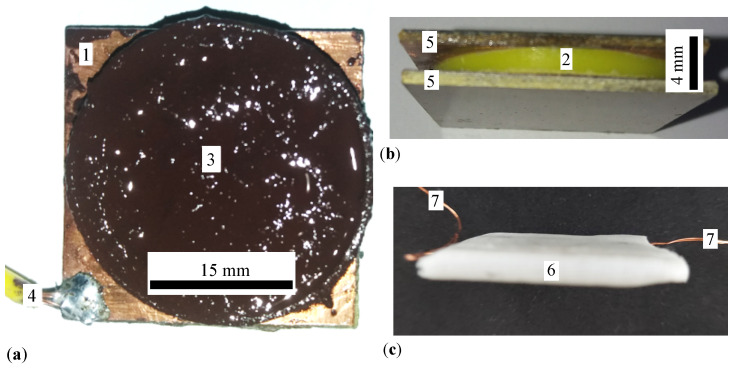
(**a**) Cylinder with CL. 1—copper foil, 3—CL, 4—electrical contact. (**b**) Cylinder with CL and with textolite plate on top. 2—dielectric ring, 5—textolite plates. (**c**) PEC. 6—adhesive medical tape. 7—terminals for electrical contact.

Two PCB boards are used to manufacture the PEC. On the copper foil (pos. 1 in Figure 1b), an insulating material ring (pos. 2 in Figure 1b) is fixed with an adhesive. At the end of this procedure, a cylinder with dimensions 28 mm × 1.4 mm is formed. In the cylinder from Figure 1b, the liquids CL_*i*_ (i=1,2,…,4) are introduced one by one. On top of the cylinder, filled with CL (Figure 2a), the copper-faced side of the second PCB board is fixed by sliding. At the end of this step, the CEE is obtained as shown in Figure 2b. This assembly is then consolidated with a medical adhesive tape (Figure 2c).

### 2.3. Experimental Setup and Measurement Protocol

**Figure 3:**
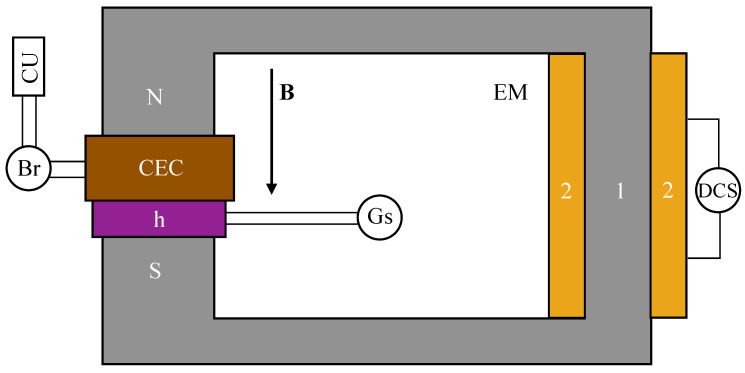
Schematic view of the experimental setup. 1—magnetic yoke, 2—coil, DCS—direct current source, Br—RLC bridge, Gs—Gauss meter, h—Hall probe, CU—computing unit, PEC—electrical capacitor based on CL, **B**—magnetic flux density vector.

The experimental setup designed for studying the susceptance and electrical conductance of PECs has the overall configuration shown in Figure 3. The setup includes a handmade electromagnet (EM) with a coil connected to a direct current source (DCS), type RXN3020D (Electronics Co Ltd., Haoxin, China). Between the N and S poles of the electromagnet, the PECs and the Hall probe (h) of the Gauss meter (GS), type DX-102 (Dexing Magnet, Xiamen, China), are fixed by turn. The PEC is connected to the RLC bridge, type CHY 41R (CHY Firemate, Tainan, Taiwan). The CHY 41R bridge, set at a frequency of 1 kHz, is used to measure the equivalent electrical capacitance and the equivalent electrical resistance of PECs, which are connected in parallel. The measurement errors of the RLC bridge are within ±1%. The measured values, taken at intervals of Δt=1 s, are transmitted and recorded by the computing unit (CU) via the RS232 interface. To check the repeatability, we performed measurements at intervals of 24 h over one week. The differences in the obtained values are ±7.15%. The observed differences are due to the sedimentation of individual particles from the oxide microfibers. These particles result from the mechanical mixing of the suspensions, which causes the breaking of the oxide microfibers.

### 2.4. Variation in Capacitance and Resistance over Time

**Figure 4:**
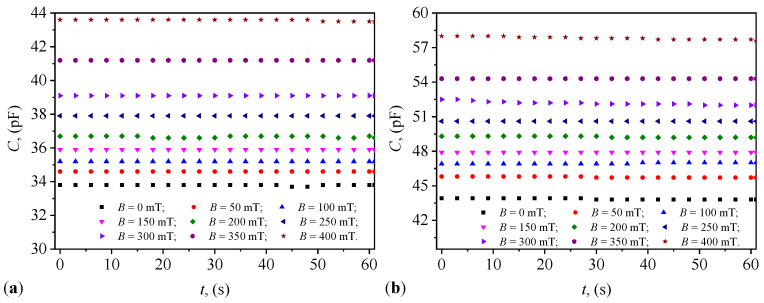

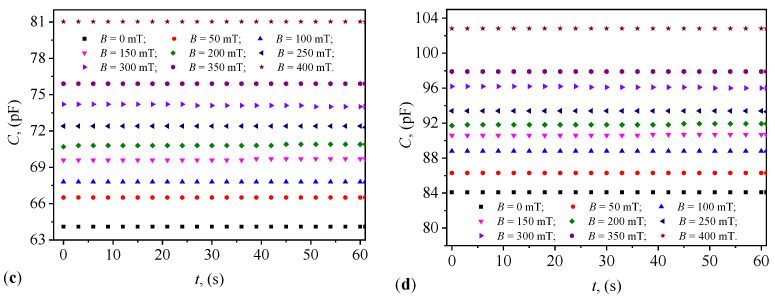
Variation in capacitance over time at different values of magnetic flux density. (**a**) PEC_1_. (**b**) PEC_2_. (**c**) PEC_3_. (**d**) PEC_4_.

**Figure 5:**
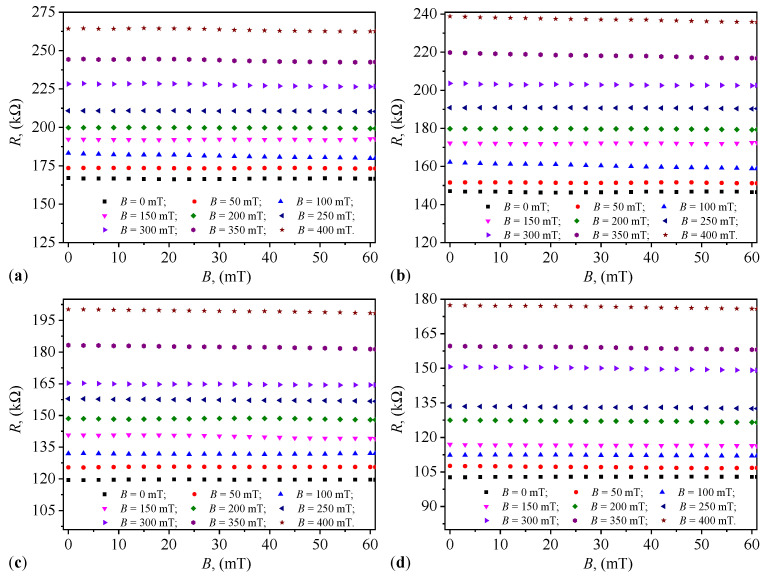
Variation in resistance over time at different values of magnetic flux density. (**a**) PEC_1_. (**b**) PEC_2_. (**c**) PEC_3_. (**d**) PEC_4_.

The variation in the equivalent capacitance, *C*, and the equivalent resistance, *R*, of the capacitors PEC_*i*_ (i=1,2,…,4) with the duration, *t*, of their maintenance at the values, *B*, of the magnetic flux density is graphically represented in Figure 4 and Figure 5. The results in Figure 4 show that the functions $C=C{\left(t\right)}_{B}$ and $R=R{\left(t\right)}_{B}$ are quasi-constant over the duration *t* of applying the value *B*. The same figures show that as the value of *B* increases, the values $C_{0_{i}}$ of the equivalent electric capacitance increase with the increase in $\Phi_{i}$ (i=1,2,…,4). Conversely, from the same figures, one can see that at the same values of $\Phi_{i}$, the values $C_{i}$ increase with the increase in *B*. Figure 5 shows that the values $R_{0_{i}}$ of the resistance decrease with the increase in $\Phi_{i}$. However, at the same values of $\Phi_{i}$ and at times *t*, one can see that the values $R_{i}$ (i=1,2,…,4) increase with the increase in *B* of the magnetic flux density.

## 3. Theoretical Model

### 3.1. Equation of Motion of Microfiber’s Microparticles in a Uniform Magnetic Field

**Figure A2:**
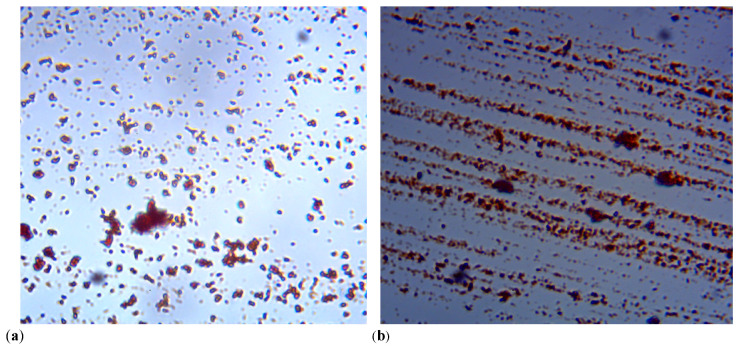
Microfibers (orange-brown aggregates) in SO (light blue). (**a**) Without any external field. (**b**) In the presence of a small magnetic field (about 20 mT).

We consider that inside the composite liquid CL, the microparticles *P* have the same diameter, equal to the average diameter $d_{m}$. At time t=0s, the PEC capacitor is introduced in a uniform magnetic field. At this point (see Figure A2a in Appendix B), the microparticles *P* transform into magnetic dipoles. The magnetic dipoles, **m**, are oriented along the direction of the Oz axis, which is identical to the direction of the magnetic flux density vector **B**. The dipoles, **m**, interact with each other, forming aggregates in the shape of columns (Figure A2b in Appendix B). These columns are uniformly distributed in the SO. The projection of the magnetic moment **m** on the direction of the Oz axis is given by [25,29]:

(1) $$m=\frac{\pi d_{m}^{3}B}{2\mu_{0}},$$

where $d_{m}$ is the average diameter, *B* is the magnetic flux density, and $\mu_{0}$ is the magnetic constant of the vacuum. At the initial moment (t=0s), the distance $\delta_{i}$ (i=1,2,3,4) between two identical and neighboring magnetic dipoles, **m**, is the same in each column. The distances $\delta_{i}$ for the composite liquids in Table 1 are calculated using the expression [25,29]:

(2) $$\delta_{i}=\frac{d_{m}}{\sqrt[{3}]{\Phi_{i}}},\;i=1,2,3,4,$$

where $d_{m}$ is the average diameter of the dipoles, **m**, and $\Phi_{i}$ is the volume fraction of the microparticles *P* in the composite liquids.

Along the Oz axis, there is a magnetic interaction between two neighboring and identical dipoles. The intensity $F_{\mathrm{mz}}$ of the dipolar magnetic interaction projected along the Oz axis is given by [25,29]:

(3) $$F_{{\mathrm{mz}}_{i}}=-\frac{3\pi \mu_{0}m^{2}}{4\pi z_{i}^{4}},$$

where *m* is the magnetic dipole moment and $z_{i}$ is the distance between the centers of mass of the magnetic dipoles at a time t>0s from the application of **B**. From Equations (1) and (3), we obtain the expression for the intensity of the interaction between two neighboring and identical dipoles in the presence of the magnetic flux density:

(4) $$F_{{\mathrm{mz}}_{i}}=-\frac{3\pi d_{m}^{6}B^{2}}{16\mu_{0}z_{i}^{4}}.$$

The microparticles, *P*, in each microfiber, are at distances $z_{i}\approx d_{m}$. Equation (4) is as follows:

(5) $$F_{{\mathrm{mz}}_{\max_{i}}}=-\frac{3\pi d_{m}^{2}B^{2}}{16\mu_{0}}.$$

The negative sign in Equation (5) indicates that the microparticles *P* attract each other.

In the time interval dt, the distance between the centers of mass of two identical and neighboring dipoles, **m**, decreases by an absolute value dz. From the composite liquid CL_*i*_, the action of $F_{{\mathrm{mz}}_{i}}$ is opposed by a resistance force $F_{{\mathrm{rz}}_{i}}$. The resistance force is the Stokes force, given by the following:

(6) $$F_{{\mathrm{rz}}_{i}}=3\pi \eta_{i}d_{m}\frac{dz_{i}}{dt},$$

where $\eta_{i}$ is the dynamic viscosity of the CL_*i*_ in the magnetic field at an arbitrary time *t*.

At an arbitrary moment *t*, a dynamic equilibrium is established, which mathematically is expressed by the equality of the two forces. Thus, we obtain the equation of motion of the dipoles, **m**, in the liquids CL_*i*_ in a magnetic field, as follows:

(7) $$\frac{dz_{i}}{dt}+\frac{d_{m}B^{2}}{16\eta_{i}\mu_{0}}=0.$$

At the moment of applying **B** (t=0s), the distance between the centers of mass of the solid microparticles in CL_*i*_ is $\delta_{i}$, and at the moment of dynamic equilibrium (t>0s), the distance between the same centers of mass is $z_{i}<\delta_{i}$. Using these conditions, we integrate Equation (7), and in the result, we introduce Equation (2), obtaining the following:

(8) $$z_{i}=\frac{d_{m}}{\sqrt[{3}]{\Phi_{i}}}\left(1-\frac{\Phi_{i}^{1/3}B^{2}}{16\mu_{0}\eta_{i}}t\right).$$

Equation (8) represents the equation of motion of the dipoles, **m**, in CL_*i*_ in a magnetic field at an arbitrary moment *t*. One can see from Equation (7) that the motion of the dipoles, **m**, in the liquids CL_*i*_ in a magnetic field is uniform. The distance between the centers of mass of the dipoles, **m**, for a fixed value of the diameter $d_{m}$ and the volume fraction of microparticles, is significantly dependent on the values of *B* of the magnetic flux density.

### 3.2. Electrical Capacitance and Resistance of PECs

**Figure 6:**
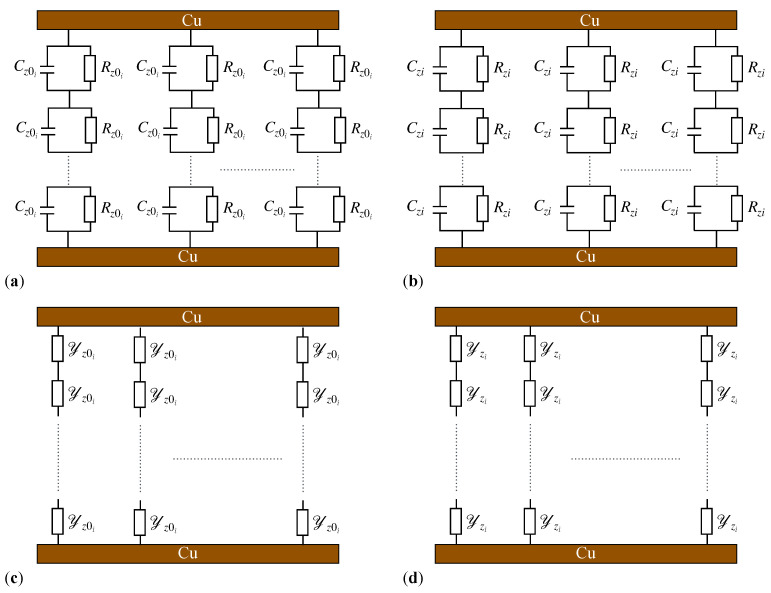
A cross-sectional view of the PEC in equivalent electrical representation. (**a**,**c**) Without a magnetic field. (**b**,**d**) With a magnetic field. $C_{{z0}_{i}}$ and $C_{z_{i}}$ are electrical micro-capacitors. $R_{{z0}_{i}}$ and $R_{z_{i}}$ are electrical micro-resistors. $Y_{{z0}_{i}}$ and $Y_{z_{i}}$ are the admittance of micro-capacitors. Cu—copper foil electrodes.

**Figure 7:**
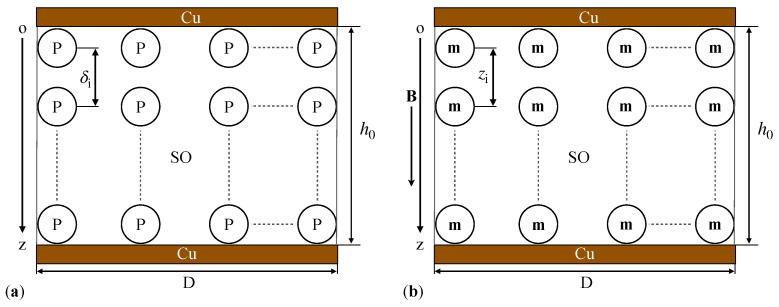
Model of a cross-sectional view of the PEC, without (**a**) and with (**b**) a magnetic field. P—iron oxide microparticles, SO—silicone oil, Cu—copper plates, D—diameter of PEC, $h_{0}$—height of PEC, **B** and **m**—magnetic flux density vector, and, respectively, dipolar electric moment, $\delta_{i}$ and $z_{i}$—distances between the center of masses of microparticles P within the CL, Oz—coordinate axis.

The results obtained in Figure 4 and Figure 5 lead us to conclude that capacitors made with the dielectric liquids CL_*i*_ (i=1,2,…,4) have an equivalent electrical circuit consisting of a resistor, R_*i*_, connected in parallel with a capacitor, C_*i*_ (Figure 6). Using the model from Figure 7, we determine the analytical expressions for the equivalent electrical resistance and capacitance.

First, we consider that the maximum number ($n_{1_{i}}$) of dipoles, **m**, in each chain is estimated by the following relation:

(9) $$n_{1_{i}}=\frac{h_{0}}{d_{m}},$$

where $h_{0}$ and $d_{m}$ are the distance between the copper electrodes of the capacitors PEC and the average diameter of the dipoles, **m**. The number ($N_{i}$) of magnetic dipoles, **m**, in the liquids (CL_*i*_) is estimated by the following relation [25,29]:

(10) $$N_{i}=\frac{\Phi_{i}V}{V_{P}},$$

where $V_{P}$ is the volume of an average particle. For $V=0.25D^{2}h_{0}$ and $V_{P}=\frac{\pi d_{m}^{3}}{6}$, substituted in Equation (10), we obtain the following:

(11) $$N_{i}=\frac{3\Phi_{i}D^{2}h_{0}}{2d_{m}^{3}}.$$

Using Equations (8) and (10), we obtain the number of columns, $n_{2_{i}}$, in the volume of the liquids CL_*i*_ as follows:

(12) $$n_{2_{i}}=\frac{N_{i}}{n_{1_{i}}}=\frac{3\Phi_{i}D^{2}}{2d_{m}^{2}},$$

Each pair of two dipoles in each chain within the liquids CL_*i*_ forms a micro-electric resistor $R_{z_{i}}$, which is electrically connected in parallel with a micro-electric capacitor $C_{z_{i}}$. We assume the micro-resistor to be linear and the micro-capacitor to be equivalent to a planar one. The resistance $R_{z_{i}}$ of the micro-resistors can be approximated by the relation $R_{z_{i}}=z_{i}/\left(\sigma_{i}S\right)$ and the capacitance $C_{z_{i}}$ of the micro-capacitors by the relation $C_{z_{i}}=\epsilon_{0}\epsilon_{i}^{\prime }S/z_{i}$, where $\sigma_{i}$ and $\epsilon_{i}^{\prime }$ are the electrical conductivity and relative dielectric permittivity of the liquids CL_*i*_ in the magnetic field, $\epsilon_{0}$ is the dielectric permittivity of the vacuum, *S* is the contact surface area between the dipoles, **m**, and $z_{i}$ is the distance between the magnetic dipoles at a time t>0s. For $S=\pi d_{m}^{2}$ and the expression of $z_{i}$ in Equation (7) introduced into the expressions for $C_{z_{i}}$ and $R_{z_{i}}$, we obtain the following:

(13) $$C_{z_{i}}=\frac{\pi d_{m}^{2}\epsilon_{0}\epsilon_{i}^{\prime }}{z_{i}}=\frac{C_{{z0}_{i}}}{1-\frac{\Phi_{i}^{1/3}B^{2}}{16\mu_{0}\eta_{i}}t},$$

where $C_{{z0}_{i}}=\pi \sqrt[{3}]{\Phi_{i}}d_{m}\epsilon_{0}\epsilon_{i}^{\prime }$, and respectively:

(14) $$R_{z_{i}}=\frac{z_{i}}{\sigma_{i}\pi d_{m}^{2}}=R_{{z0}_{i}}\left(1-\frac{\Phi_{i}^{1/3}B^{2}}{16\mu_{0}\eta_{i}}t\right),$$

where $R_{{z0}_{i}}={\left(\pi d_{m}\sigma_{i}\sqrt[{3}]{\Phi_{i}}\right)}^{-1}$. The equivalent electrical representations in the absence and the presence of a magnetic field are shown in Figure 6a,b, respectively.

The capacitors $C_{z_{i}}$ in the chains of magnetic dipoles are connected in series, and their number is $n_{1_{i}}\gg 1$. Then, the capacitance $C_{z_{1_{i}}}$ of a chain of capacitors is estimated by the following relation:

(15) $$C_{z_{1_{i}}}=\frac{C_{z_{i}}}{n_{1_{i}}}=\frac{\pi d_{m}^{2}\epsilon_{0}\epsilon_{i}^{\prime }\sqrt[{3}]{\Phi_{i}}}{h_{0}\left(1-\frac{\Phi_{i}^{1/3}B^{2}}{16\mu_{0}\eta_{i}}t\right)},$$

The capacitors $C_{z_{1_{i}}}$ are connected in parallel through the copper foil of the capacitors PEC_*i*_ (i=1,2,…,4) in the magnetic field. Then, the equivalent capacitance $C_{i}$ of the capacitors is given by the following:

(16) $$C_{i}=n_{2_{i}}C_{z_{1_{i}}}=\frac{3\pi D^{2}\epsilon_{0}\epsilon_{i}^{\prime }\Phi_{i}^{4/3}}{2h_{0}\left(1-\frac{\Phi_{i}^{1/3}B^{2}}{16\mu_{0}\eta_{i}}t\right)}=\frac{C_{0_{i}}}{1-\frac{\Phi_{i}^{1/3}B^{2}}{16\mu_{0}\eta_{i}}t},$$

where $C_{0_{i}}$ is the initial capacitance of the capacitors PEC_*i*_.

The value of $C_{0_{i}}$ is calculated using the following relation:

(17) $$C_{0_{i}}=\frac{3\pi D^{2}\epsilon_{0}\epsilon_{i}^{\prime }\Phi_{i}^{4/3}}{2h_{0}},$$

The equivalent resistance of a chain of magnetic dipoles, in number $n_{1_{i}}\gg 1$, is formed by the sum of the resistances in each chain of magnetic dipoles, and is given by the following:

(18) $$R_{z_{1_{i}}}=n_{1_{i}}R_{z_{i}}=\frac{h_{0}}{\sigma_{i}\pi d_{m}^{2}\sqrt[{3}]{\Phi_{i}}}\left(1-\frac{\Phi_{i}^{1/3}B^{2}}{16\mu_{0}\eta_{i}}t\right),$$

The resistances $R_{z_{1_{i}}}$ (i=1,2,…,4) are connected in parallel through the copper foil of the capacitors PEC_*i*_ in the magnetic field. Then, the equivalent resistance $R_{i}$ of the capacitors is estimated by the following relation:

(19) $$R_{i}=\frac{R_{z_{1_{i}}}}{n_{2_{i}}}=\frac{2h_{0}}{3\pi D^{2}\sigma_{i}\Phi_{i}^{4/3}}\left(1-\frac{\Phi_{i}^{1/3}B^{2}}{16\mu_{0}\eta_{i}}t\right)=R_{0_{i}}\left(1-\frac{\Phi_{i}^{1/3}B^{2}}{16\mu_{0}\eta_{i}}t\right),$$

where $R_{0_{i}}$ are the initial equivalent electrical resistances of the capacitors PEC_*i*_. The value of $R_{0_{i}}$ is calculated using the following relation:

(20) $$R_{0_{i}}=\frac{2h_{0}}{3\pi D^{2}\sigma_{i}\Phi_{i}^{4/3}}.$$

Note that the resistance in Figure 5 increases with the increase in *B*, which is contrary to Equation (19). This discrepancy is due to the hematite in the composition of the microfibers. It is known that hematite nano-microparticles polarize in the Earth’s magnetic field. By applying an external magnetic field, the hematite nano-microparticles form chains between the magnetite nano-microparticles through magnetic polarization. The length of these chains increases with the increase in *B* of the magnetic flux density. It is known that hematite nano-microparticles are iron oxide semiconductors with much lower electrical conductivity compared to magnetite nano-microparticles. The effect is an increase in the equivalent electrical resistance of the capacitors PEC_*i*_ with the increase in *B* of the magnetic flux density, contrary to the effects observed in liquid composites based on carbonyl iron microparticles [25,27,29].

In addition, upon the application of the electric field, the microfibers become polarized. Through this polarization and friction, there is an increase in the amount of electric charge. By decreasing the distance, the capacitance of the micro-capacitors within the dielectric volume between the plates of the plane capacitor increases. Thus, overall, there is an increase in the equivalent electrical capacitance and a decrease in the equivalent electrical resistance of the PECs.

### 3.3. Susceptance and Conductance of PECs

Generally, one can write the susceptance B and electrical conductance G of the capacitors as the imaginary, and, respectively, the real part of the admittance, i.e.,

(21) Y=G+jB.

For the PEC_*i*_ (i=1,2,3,4) investigated here, and in the absence of the magnetic field, we consider that each $\left(C_{{z0}_{i}},R_{{z0}_{i}}\right)$ pair shown in Figure 6a has a corresponding admittance $Y_{{z0}_{i}}$, as shown in Figure 6c. Then, for the total admittance $Y_{0_{i}}$ of the PEC_*i*_, Equation (21) is as follows:

(22) $$Y_{0_{i}}=G_{0_{i}}+jB_{0_{i}},$$

where $G_{0_{i}}=1/R_{0_{i}}$, $B_{0_{i}}=2\pi fC_{0_{i}}$. Here, $R_{0_{i}}$ and $C_{0_{i}}$ are given by Equation (20), and, respectively, (17).

In the presence of the magnetic field, we consider that each $\left(C_{z_{i}},R_{z_{i}}\right)$ pair in Figure 6b has a corresponding admittance $Y_{z_{i}}$, as shown in Figure 6d. Then, for the total admittance $Y_{i}$ of the PEC_*i*_, Equation (21) is as follows:

(23) $$Y_{i}=G_{i}+jB_{i}.$$

Here, the conductance $G_{i}$ of the capacitors PEC_*i*_ is defined by the following relation:

(24) $$G_{i}=\frac{1}{R_{i}}=\frac{G_{0_{i}}}{1-\frac{\Phi_{i}^{1/3}B^{2}}{16\mu_{0}\eta_{i}}t},$$

and the susceptance $B_{i}$ of the capacitors PEC_*i*_ is defined by the following relation:

(25) $$B_{i}=2\pi fC_{i}=\frac{2\pi fC_{0_{i}}}{1-\frac{\Phi_{i}^{1/3}B^{2}}{16\mu_{0}\eta_{i}}t}=\frac{B_{0_{i}}}{1-\frac{\Phi_{i}^{1/3}B^{2}}{16\mu_{0}\eta_{i}}t}.$$

## 4. Experimental Results

The PEC is placed between the N and S poles of the electromagnet shown in Figure 3. Using measurements from the h connected to the DX-102 Gauss meter, the magnetic flux densities are adjusted to values B≤0.2 mT. The terminals of the PEC are connected to the input of the CHY 41R bridge, set at a frequency of f=1 kHz. The equivalent electrical capacitance *C* and the equivalent electrical resistance *R* are measured and recorded, considering the electrical representation of the PECs as an electric dipole consisting of an ideal capacitor connected in parallel with an ideal resistor. Measurements are recorded at time intervals Δt=1 s for 60 s, for magnetic flux density value *B* adjusted in steps of ΔB=50 mT up to a maximum of 400 mT. After recording the values of *C* and *R*, the magnetic flux density *B* is adjusted to a new value, without reverting to the initial value, and measurements of *C* and *R* continue at one-second intervals until the 60-s period is exhausted. This procedure is then repeated.

**Figure 8:**
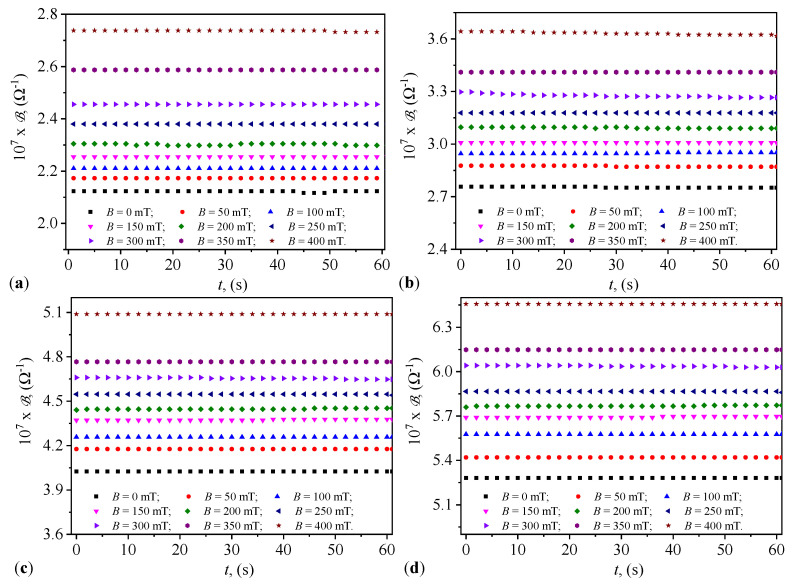
Variation with time of the susceptance of PECs, at different values of the magnetic flux density. (**a**) PEC_1_. (**b**) PEC_2_. (**c**) PEC_3_. (**d**) PEC_4_.

**Figure 9:**
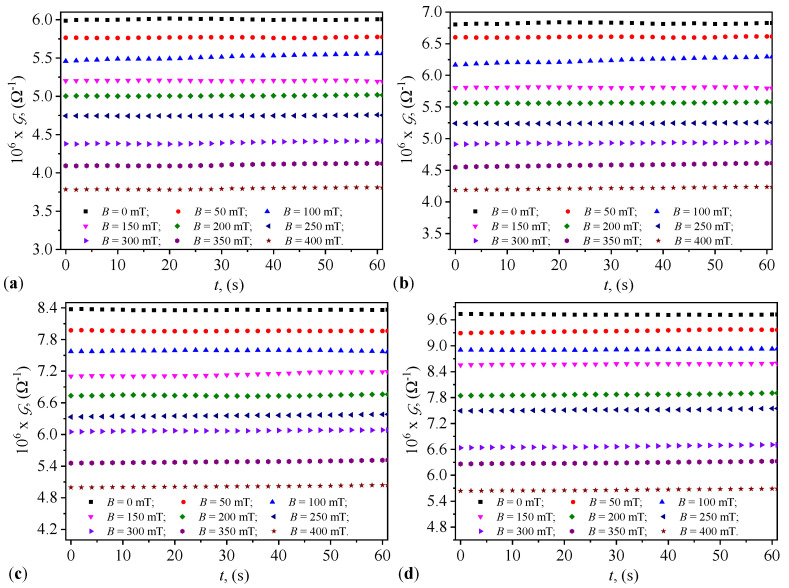
Variation with time of the conductance of PECs, at different values of the magnetic flux density. (**a**) PEC_1_. (**b**) PEC_2_. (**c**) PEC_3_. (**d**) PEC_4_.

Knowing that the electrical susceptance and conductance of the PECs are defined by the relationships B=2πfC and, respectively, G=1/R, then using the functions $C=C{\left(t\right)}_{B}$ and $R=R{\left(t\right)}_{B}$ from Figure 4 and, respectively, Figure 5) we obtain the variations $B=B{\left(t\right)}_{B}$ and $G=G{\left(t\right)}_{B}$, graphically represented in Figure 8 and Figure 9.

It can be observed from these figures that the quantities B and G are stable over time. This stability is related to the fact that during the preparation of the CLs, nano-microparticles of hematite, maghemite, and magnetite detach from the mFe microfibers. The hematite nano-microparticles instantly polarize magnetically and form stable aggregates in the absence of the magnetic field [32,33]. These aggregates, based on reports in [32], exhibit high friction when moving in the SO, thus eliminating or at least reducing the sedimentation of the solid phase. This effect is observed by noting that the values B and the G are stable over time. Figure 8 and Figure 9 also show that the quantities B and G increase with the increasing values of Φ in the CLs of PECs.

On the other hand, while the functions $B=B_{B}$ increase with the increasing values *B* of the magnetic flux density in accordance with Equation (25), the functions $G=G{\left(t\right)}_{B}$ show values that decrease with the increasing values of the same magnetic flux density, contrary to Equation (24). This discrepancy is due to the semiconductor properties of the hematite nano-microparticles [34]. As the values of the magnetic flux density increase, the thickness of the layer formed by hematite nano-microparticles in the vicinity of the magnetite and maghemite microparticles also increases. This results in reduced electrical conductivity and an increase in the relative dielectric permittivity of the liquids, due to the concentration of electric charges within the volume of the CLs. The observed effects are the increase in the value of B and the decrease in the values of G with the increasing magnetic flux density.

**Figure 10:**
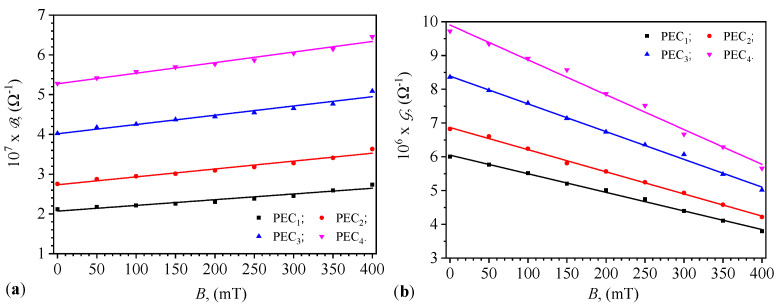
Variation with magnetic flux density of the susceptance (**a**) and conductance (**b**) for PECs.

Using the functions $B=B{\left(t\right)}_{B}$ from Figure 8 for 0≤t(s)≤60, the average values $B_{m}$ are calculated as a function of the values *B* of the magnetic flux density. The functions $B_{m}=B_{m}{\left(B\right)}_{{\mathrm{PEC}}_{i}}$, for i=1,2,3,4, are obtained and shown in Figure 10a. Proceeding identically, but using the functions $G=G{\left(t\right)}_{B}$ from Figure 9, the functions $G_{m}=G_{m}{\left(B\right)}_{{\mathrm{PEC}}_{i}}$ are obtained and shown in Figure 10b. In both cases, the errors are within ±1%, excepting the case of conductance of PEC_1_ at B=100 mT, and where the error is ±2.89%.

One can see from Figure 10a that the functions $B_{m}=B_{m}{\left(B\right)}_{{\mathrm{PEC}}_{i}}$ have the following form:

(26) $$B_{m}=B_{0}+\alpha_{B}\cdot B,$$

where $B_{0}$ is the initial electric susceptibility and $\alpha_{B}$ is the slope. By fitting data in Figure 10a with Equation (26), one obtains the values of the parameters $B_{0}$ and $\alpha_{B}$, as listed in Table 2.

From Figure 10b, we observe that the functions $G_{m}=G_{m}{\left(B\right)}_{{\mathrm{PEC}}_{i}}$ have the following form:

(27) $$G_{m}=G_{0}-\alpha_{G}\cdot B,$$

where $G_{0}$ is the initial electric conductance and $\alpha_{G}$ is the slope. By fitting data in Figure 10b with Equation (27), one obtains the values of the parameters $G_{0}$ and $\alpha_{G}$, as listed in Table 2.

It can be observed from Figure 8 that the dependence of the quantities $B_{m}$ and $G_{m}$ on the values *B* of the magnetic flux density is quasi-constant, in accordance with Equation (25). This result is due to hematite nano-microparticles which form aggregates that cannot be broken down by thermal energy [32,33,34]. On the other hand, the viscosity $\eta_{i}$ of the liquids CL_*i*_ (i=1,2,3,4) increases with the increasing values of *B* of the magnetic flux density, accompanied by the formation of new aggregate structures [32,33]. From Figure 10, we observe that the values of the quantities $B_{m}$ and $G_{m}$ of the capacitors PEC_*i*_ are coarsely adjusted by the choice of liquids CL_*i*_ and finely by the values of *B* of the magnetic flux density.

The dynamic viscosity $\eta_{i}$ of the liquids CL_*i*_ (i=1,2,3,4) in the absence of a magnetic field can be approximated using the following relation [35,36]:

(28) $$\eta_{i}=\eta_{SO}\left(1+2.5\Phi_{i}+6.4\Phi_{i}^{2}\right),$$

where $\eta_{SO}=0.97\;\mathrm{Pa}\cdot s$ is the dynamic viscosity of SO at a temperature of 24 °C. For the volume fractions $\Phi_{i}$ given in Table 1, the dynamic viscosity of the liquids CL_*i*_ in the absence of a magnetic field has the following values:

(29) $$\eta \left(\mathrm{Pa}\times s\right)=\left\{\begin{array}{cc} 1.70332, & \mathrm{for}\;\Phi_{1}=20\;\mathrm{vol}.\%\\ 2.93328, & \mathrm{for}\;\Phi_{2}=40\;\mathrm{vol}.\%\\ 4.65988, & \mathrm{for}\;\Phi_{3}=60\;\mathrm{vol}.\%\\ 6.88312, & \mathrm{for}\;\Phi_{4}=80\;\mathrm{vol}.\% \end{array}\right.$$

From the set of values in Equation (28), it can be observed that the dynamic viscosity increases with the increase in the volume fractions of microfibers.

It is well-known that in a magnetic field, the solid phase, in the form of ferri-ferromagnetic nano-microparticles, forms aggregates within the liquid matrix [25,28,29]. This effect transforms the liquid from Newtonian to non-Newtonian [37,38]. To determine the viscosity of the liquids in a magnetic field, we use Equation (25), from which we obtain the following:

(30) $$\eta_{B_{i}}=\frac{\Phi_{i}^{1/3}B^{2}t}{16\mu_{0}\left(1-\frac{B_{0_{i}}}{B_{m_{i}}}\right)},\;i=1,2,3,4,$$

where $B_{0_{i}}$ and $B_{m_{i}}$ are the average values of the susceptance of the capacitors PEC_*i*_ at the initial moment and at time *t*, respectively. The latter refers to the average duration for which the capacitors PEC_*i*_ are maintained in the magnetic field (see above).

For t=30s, $\mu_{0}=4\pi \times 10^{-7}\;H/m$, and the values of $\Phi_{i}$ (i=1,2,…,4) from Table 1, when substituted in Equation (30), we obtain the following expressions for the viscosity of the liquids CL_*i*_ in a magnetic field:

(31) $$\eta_{B_{i}}=\left\{\begin{array}{cc} \frac{0.873B^{2}\;\left(\mathrm{mT}\right)}{1-\frac{B_{0_{1}}}{B_{m_{1}}}}, & \mathrm{for}\;\Phi_{1}=20\;\mathrm{vol}.\%\\ \frac{1.010B^{2}\;\left(\mathrm{mT}\right)}{1-\frac{B_{0_{2}}}{B_{m_{2}}}}, & \mathrm{for}\;\Phi_{2}=40\;\mathrm{vol}.\%\\ \frac{1.259B^{2}\;\left(\mathrm{mT}\right)}{1-\frac{B_{0_{3}}}{B_{m_{3}}}}, & \mathrm{for}\;\Phi_{3}=60\;\mathrm{vol}.\%\\ \frac{1.386B^{2}\;\left(\mathrm{mT}\right)}{1-\frac{B_{0_{4}}}{B_{m_{4}}}}, & \mathrm{for}\;\Phi_{4}=80\;\mathrm{vol}.\% \end{array}\right.$$

**Figure 11:**
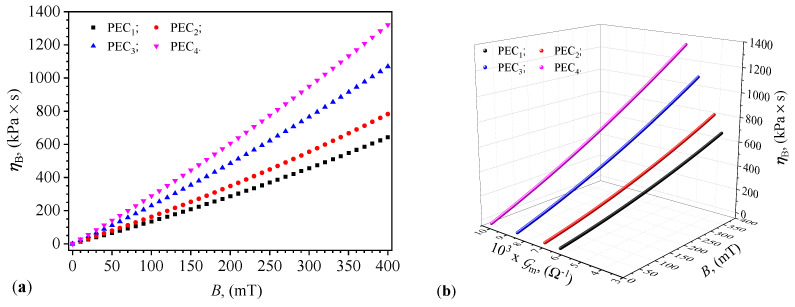
Variation of viscosity $\eta_{B}$ with magnetic flux density (**a**), and with magnetic flux density and conductance (**b**) for PECs.

In these expressions, we substitute the functions $B_{m}=B_{m}{\left(B\right)}_{{\mathrm{PEC}}_{i}}$ (i=1,2,…,4) from Figure 10a, and obtain the functions $\eta_{B_{i}}=\eta_{B_{i}}{\left(B\right)}_{{\mathrm{PEC}}_{i}}$ as shown in Figure 11a. It can be observed from this figure and the group of values in Equation (28) that the viscosity $\eta_{B}$ increases by up to three orders of magnitude in a magnetic field and remains stable over time, as the liquids CL_*i*_ do not sediment. From the same Figure 11a, one can see that the viscosity $\eta_{B}$ of the liquids depends on the amount of magnetizable phase used and is significantly influenced by the values of *B* of the magnetic flux density. The obtained results are due to the formation of aggregates in the SO, an effect also demonstrated in Refs. [37,38]. In Ref. [37], a composite liquid consisting of SO with carbonyl iron microparticles stabilized with silicon nanoparticles is used. The dynamic viscosities of these composites, as with those in the present work, depend on the volume fractions of the magnetizable phase and stabilizing additives, and are significantly influenced by the magnetic field. The values obtained for the dynamic viscosity with these composite liquids are comparable to those in Figure 11a. Ref. [38] reports an extensive study on the stability of magnetizable composite liquids. This study discusses preparation methods based on carbonyl iron microparticles, SO, and additives. The results regarding the rheological properties of the composite liquids are remarkable and comparable to those in Figure 11a, but obtained through a multi-phase technological process.

**Figure 12:**
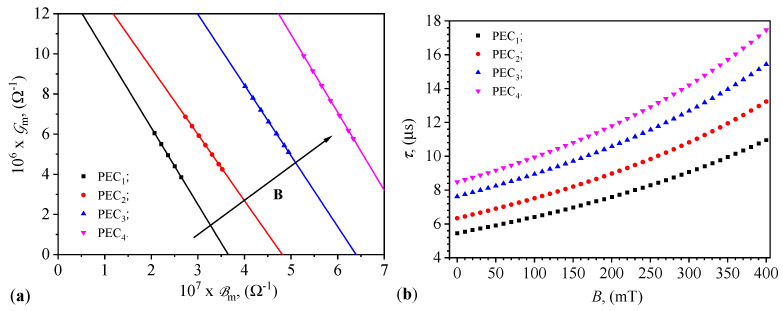
(**a**) Variation of average conductance $G_{m}$ with average susceptance $B_{m}$ for PECs. (**b**) Variation in the time constant with magnetic flux density for PECs. The black arrow indicates the direction of increase of the magnetic field density in the $\left(G_{m},B_{m}\right)$ plane.

The quantities $\eta_{B}$ and $G_{m}$ share the same feature, namely they describe CL_*i*_ in the capacitors PEC_*i*_ (i=1,2,3,4) subjected to a magnetic field. Hence, the natural relationship between $\eta_{B}$, $G_{m}$, and the values *B* of the magnetic flux density is depicted in Figure 11b. This shows that from the conductance measurements corresponding to the values *B* of the magnetic flux density, the values of $\eta_{B}$ for the magnetically active CL_*i*_ can be determined. The functions $G_{m}=G_{m}{\left(B\right)}_{{\mathrm{PEC}}_{i}}$ and $B_{m}=B_{m}{\left(B\right)}_{{\mathrm{PEC}}_{i}}$, describe physical mechanisms occurring on the same basis, namely the liquids CL_*i*_. Thus, there are correlations between these functions, as shown in Figure 12a. These results demonstrate that by choosing the composition of the liquids CL_*i*_ the operating points ($B_{m},G_{m}$) of the capacitors PEC_*i*_ can be coarsely adjusted. In contrast, by selecting the values of *B* of the magnetic flux density, the values of $B_{m}$ and $G_{m}$ can be finely tuned. For very high values of *B* the microfibers form compact aggregates and the capacitor becomes a resistor. Thus, in this case, the proposed model cannot be applied.

Given the functions $B_{m}=B_{m}{\left(B\right)}_{{\mathrm{PEC}}_{i}}$ from Figure 10a and $G_{m}=G_{m}{\left(B\right)}_{{\mathrm{PEC}}_{i}}$ from Figure 10b, we define the time constant τ of the capacitors PEC_*i*_ (i=1,2,…,4) using the following expression:

(32) $$\tau =\frac{B_{m}}{2\pi fG_{m}},$$

where *f* is the frequency of the alternating electric field. By substituting the functions from Figure 10a,b in Equation (32) and setting f=1kHz, we obtain the functions $\tau =\tau {\left(B\right)}_{{\mathrm{PEC}}_{i}}$ shown in Figure 12b. It can be observed from this figure that the values of τ can be coarsely adjusted by selecting the ratio of mFe microfibers to SO, and finely tuned by the values of *B* of the magnetic flux density. This result leads us to conclude that the capacitors PEC_*i*_ are useful for creating magnetically controlled time relays and, in a medium-frequency electric field, useful for automating technological processes. A practical application in such processes is optimizing the response speed of electronic components to ensure timely and accurate control actions. The ability to adjust the time constant through the ratio of susceptance to conductance, as given by Equation (32) (see also Figure 12b), provides flexibility to tune the system’s response according to the specific requirements of the application. Applications requiring quick response times (e.g., real-time control systems) would benefit from a lower time constant. This can be achieved by increasing the conductance or decreasing the susceptance. However, applications that prioritize stability and noise immunity over rapid response might require a higher time constant. This can be managed by decreasing the conductance or increasing the susceptance.

## 5. Discussions

The investigation into the electrical behavior of plane capacitors utilizing magnetodielectric materials composed of magnetizable microfibers dispersed within an SO matrix has yielded several significant findings. These results have important implications for the design and optimization of capacitors for applications in microelectronics and sensor technologies.

Figure 8 and Figure 9 illustrate the variation in electrical susceptance and conductance with time under different magnetic flux densities for the four different volume concentrations of microfibers in the capacitors. The susceptance increased over time, indicating the capacitors’ ability to dynamically adjust their electrical properties in response to external magnetic fields, consistent with trends reported in studies involving magnetodielectric composites (Andrei et al. [25]). Conversely, conductance decreased with increasing magnetic flux density, highlighting the magnetic field’s influence in controlling conductive pathways within the dielectric medium, as observed by Iacobescu et al. [29]. The temporal stability of both conductance and susceptance suggests that the capacitors maintain consistent performance under continuous exposure to the magnetic field, essential for reliable operation in practical applications.

Figure 10 demonstrates the variation in susceptance and conductance with magnetic flux density, where susceptance showed a linear increase, affirming the magnetic field’s effectiveness in enhancing the dielectric properties of the capacitors, consistent with the dipolar approximation model by Bica et al. [39]. Conversely, conductance exhibited a linear decrease with increasing magnetic flux density, attributed to the formation of chain-like structures of microfibers under the magnetic field, which increases the dielectric constant while reducing overall conductivity due to decreased mobility of charge carriers. These effects have potential applications in designing tunable electronic components, such as adaptive filters and sensors, which can benefit from dynamically adjustable electrical properties.

The variation in viscosity with magnetic flux density, as shown in Figure 11, provides further insights into the internal dynamics of the magnetodielectric materials. The increase in viscosity with magnetic flux density is consistent with the aggregation of iron oxide microfibers, forming stable structures that resist shear flow. This transformation from a Newtonian to a non-Newtonian fluid under the influence of a magnetic field has significant implications for the mechanical stability and performance of the capacitors. This behavior is well-documented in the rheological studies of magnetic fluids by Wu et al. [40], where the magnetic field induces the formation of chain-like structures, increasing the fluid’s viscosity. Additionally, the correlation between viscosity, magnetic flux density, and conductance underscores the complex interplay between mechanical and electrical properties in these composite materials, thereby enhancing their application potential in adaptive systems.

The linear relationship observed between the average conductance and average susceptance with magnetic flux density in Figure 12, confirms the predictable nature of the capacitors’ performance under varying magnetic fields. This predictability is crucial for the practical application of these capacitors in real-world systems, as highlighted by Zhang et al. [41], who demonstrated the importance of stable and predictable electrical properties in the development of smart electronic components. The time constant, which can be adjusted by changing the ratio of microfibers to SO and the magnetic flux density, highlights the potential for fine-tuning the response time of these capacitors for specific applications.

The tunability of the electrical characteristics of the capacitors, achieved by adjusting the fiber content of the SO and the values of the magnetic flux density, can be harnessed in several advanced technologies. Specifically, these capacitors can transition between capacitive, resistive, or a combination of both properties, making them highly versatile for various applications, such as:

- Automation systems: In automation blocks, the ability to dynamically adjust the capacitive or resistive properties of the capacitors remotely can enhance the functionality and efficiency of control modules.
- Electrical circuits: The magnetic programmability of the capacitive or resistive character of the capacitors is especially beneficial in electrical circuits. This feature enables the design of circuits with adjustable electrical properties, improving the performance and flexibility of electronic components used in communication devices, signal processing, and other electronic applications.
- Field sensors for medical devices: The capacitors’ tunable properties also make them suitable for use as field sensors in medical devices, particularly for individuals with pacemakers. The capacitors can detect and respond to external magnetic fields, providing critical data on environmental conditions that could affect the operation of pacemakers. This capability enhances the safety and reliability of medical monitoring systems, ensuring better protection for patients.

## 6. Conclusions

In this study, we successfully fabricated electrical capacitors using cost-effective materials, specifically a composite liquid synthesized from silicone oil (SO) and iron oxide microfibers (hematite, maghemite, and magnetite) at high temperatures. The presence of hematite, a semiconductor iron oxide with spontaneous magnetization, was pivotal in forming stable aggregates with maghemite and magnetite nano-microparticles. These aggregates, characterized by low mass density and high roughness, resisted sedimentation, ensuring the long-term stability of the capacitors’ electrical properties.

Our findings revealed that the susceptance and electrical conductance of these capacitors remained stable over extended periods. Unlike traditional capacitors utilizing carbonyl iron microparticles, our capacitors demonstrated a unique behavior where electrical conductance decreased with increasing magnetic flux density. This phenomenon is attributed to the semiconductor properties of hematite, which differs significantly from the behavior of conventional magnetizable composite liquids where conductance typically increases with magnetic field strength.

The dynamic viscosity of the composite liquids also increased notably in the presence of a magnetic field, similar to classical magnetizable composites. The major advantage of our proposed method lies in its single-phase, cost-efficient production process for these composite liquids. The linear dependency of susceptance and conductance values observed in the developed capacitors suggests their potential application in electrical equipment designed for testing rheological properties and control blocks.

The capacitors’ predictable performance under varying magnetic fields underscores their practical applicability in real-world systems, particularly in automation technologies. The ability to fine-tune the capacitors’ response time by adjusting the microfiber-to-SO ratio and magnetic flux density is especially valuable. These attributes make the capacitors suitable for developing advanced systems with enhanced performance characteristics, such as adaptive sensors and electronic components with dynamically adjustable properties.

Our research contributes significantly to the understanding and advancement of magnetodielectric-based capacitors, offering insights into their design and optimization for various applications in microelectronics and sensor technologies. The theoretical model based on dipolar approximations provided a robust framework to explain the observed phenomena, further validating our experimental results.

In conclusion, the innovative use of iron oxide microfibers in an SO matrix presents a promising avenue for developing high-performance, cost-effective capacitors. Indeed, several issues related to the proposed technology need to be addressed. The primary concerns involve the number of microfibers and the relative content of hematite, maghemite, and magnetite. Higher quantities of microfibers can lead to sedimentation issues, but this can be resolved by incorporating the suspension into a high-viscosity liquid polymer matrix or by embedding the microfibers in polymerized silicone rubber. These approaches ensure stability over several years. The relative content of the oxides is crucial. A higher magnetite content increases magnetic responsiveness, enhancing the material’s performance in magnetic applications. Conversely, a high hematite content, due to its semiconducting properties, might affect the electroconductive properties, potentially limiting the ability to fine-tune the response times of the composite and adapt to varying magnetic fields. This adaptability is essential for applications in advanced systems and automation technologies. Therefore, developing a technological process that allows precise control over the relative content of oxides and utilizing an appropriate embedding matrix is essential to maintain both electrical performance and overall stability. Future research should focus on refining these aspects to ensure the capacitors’ long-term stability and scalability in various conditions and applications.

## Figures and Tables

**Table 1:** The composition of CLs and their saturation magnetization ($\sigma_{s_{\mathrm{CL}}}$). $\Phi_{\mathrm{mFe}}$ and $\phi_{\mathrm{mFe}}$ are the volume, and, respectively, mass fractions of mFe.

	mFe, (cm^3^)	mFe, (g)	SO, cm^3^)	SO, (g)	$\Phi_{\mathrm{mFe}}$, (vol.%)	$\phi_{\mathrm{mFe}}$, (wt.%)	$\sigma_{\mathrm{sCL}}$, (A·m^2^/kg)
CL_1_	0.20	0.575	1.80	1.75	10	24.8	2.27
CL_2_	0.40	1.150	1.60	1.55	20	42.6	4.54
CL_3_	0.60	1.725	1.40	1.36	30	56.0	6.81
CL_4_	0.80	2.300	1.20	1.16	40	66.4	9.08

**Table 2:** The values of the parameters $B_{0}$, $\alpha_{B}$, $G_{0}$ and $\alpha_{G}$ in Equations (26) and (27).

	$B_{0}$, ($\Omega^{-1}$)	$\alpha_{B}$, ($\Omega^{-1}$/mT)	$G_{0}$, ($\Omega^{-1}$)	$\alpha_{G}$, ($\Omega^{-1}$/mT)
PEC_1_	2.070 ± 0.034	0.001 ± 1.409 × 10^−4^	6.049 ± 0.030	0.006 ± 1.188 × 10^−4^
PEC_2_	2.733 ± 0.034	0.002 ± 1.412 × 10^−4^	6.867 ± 0.030	0.007 ± 1.166 × 10^−4^
PEC_3_	4.016 ± 0.043	0.002 ± 1.785 × 10^−4^	6.388 ± 0.042	0.008 ± 1.757 × 10^−4^
PEC_4_	5.273 ± 0.039	0.003 ± 1.617 × 10^−4^	9.901 ± 0.092	0.010 ± 3.849 × 10^−4^

## Data Availability

The original contributions presented in this study are included in the article; further inquiries can be directed to the corresponding author.

## Appendix A. Morphology of Iron Oxide Microfibers

The morphology of the iron oxide microfibers was obtained using an Inspect S (SEM) electron microscope from FEI Europe B.V., Eindhoven, The Netherlands. The image of the microfibers obtained with this microscope is shown in Figure A1a. From this photograph, one can see that the nano-microfibers are composed of chained nano-microparticles. In Figure A1b, 200 nano-microparticles are selected, marked in red, and their dimensions are measured. The size distribution of the nano-microparticles in the microfibers is shown in Figure A1c. A fit (red curve) with a Gaussian function is performed, and the obtained average diameter of the microparticles in the mFe microfibers is $d_{m}=0.94\;\mu m\pm 0.01\;\mu m$.

## Appendix B. Arrangement of Microfibers without and with an External Magnetic Field

In the absence of any external field, the microfibers form aggregates randomly dispersed within the SO matrix, as shown in Figure A2a. When an external field is applied, the aggregates are arranged in chain-like structures, as shown in Figure A2b.

## References

[1] Ariyarathna T., Kularatna N., Gunawardane K., Jayananda D., Steyn-Ross D.A. (2021). Development of Supercapacitor Technology and Its Potential Impact on New Power Converter Techniques for Renewable Energy. IEEE J. Emerg. Sel. Top. Ind. Electr..

[2] Subasinghage K., Gunawardane K., Padmawansa N., Kularatna N., Moradian M. (2022). Modern Supercapacitors Technologies and Their Applicability in Mature Electrical Engineering Applications. Energies.

[3] Laadjal K., Cardoso A.J.M. (2023). Multilayer Ceramic Capacitors: An Overview of Failure Mechanisms, Perspectives, and Challenges. Electronics.

[4] Abid D., Mjejri I., Oueslati A., Guionneau P., Pechev S., Daro N., Elaoud Z. (2024). A Nickel-Based Semiconductor Hybrid Material with Significant Dielectric Constant for Electronic Capacitors. ACS Omega.

[5] Chaban V.V., Andreeva N.A. (2024). Higher hydrogen fractions in dielectric polymers boost self-healing in electrical capacitors. Phys. Chem. Chem. Phys..

[6] Yadlapalli R.T., Alla R.R., Kandipati R., Kotapati A. (2022). Super capacitors for energy storage: Progress, applications and challenges. J. Energy Storage.

[7] Torki J., Joubert C., Sari A. (2023). Electrolytic capacitor: Properties and operation. J. Energy Storage.

[8] He Q., Sun K., Shi Z., Liu Y., Fan R. (2023). Polymer dielectrics for capacitive energy storage: From theories, materials to industrial capacitors. Mater. Today.

[9] Wang C., Ali L., Meng F.Y., Adhikari K.K., Zhou Z.L., Wei Y.C., Zou D.Q., Yu H. (2021). High-Accuracy Complex Permittivity Characterization of Solid Materials Using Parallel Interdigital Capacitor- Based Planar Microwave Sensor. IEEE Sens. J..

[10] Sun Z., Wen X., Wang L., Yu J., Qin X. (2022). Capacitor-inspired high-performance and durable moist-electric generator. Energy Environ. Sci..

[11] Ma Z., Zhang Y., Zhang K., Deng H., Fu Q. (2023). Recent progress in flexible capacitive sensors: Structures and properties. Nano Mater. Sci..

[12] Zhou B., Dong Y., Chi Q., Zhang Y., Chang L., Gong M., Huang J., Pan Y., Wang X. (2020). Fe-based amorphous soft magnetic composites with SiO2 insulation coatings: A study on coatings thickness, microstructure and magnetic properties. Ceram. Int..

[13] Pawar S.G., Pradnyakar N.V., Modak J.P. (2021). Piezoelectric transducer as a renewable energy source: A review. J. Phys. Conf. Ser..

[14] Gemelli A., Tambussi M., Fusetto S., Aprile A., Moisello E., Bonizzoni E., Malcovati P. (2023). Recent Trends in Structures and Interfaces of MEMS Transducers for Audio Applications: A Review. Micromachines.

[15] Zhang T., Sun H., Yin C., Jung Y.H., Min S., Zhang Y., Zhang C., Chen Q., Lee K.J., Chi Q. (2023). Recent progress in polymer dielectric energy storage: From film fabrication and modification to capacitor performance and application. Progr. Mater. Sci..

[16] Huang S., Liu K., Zhang W., Xie B., Dou Z., Yan Z., Tan H., Samart C., Kongparakul S., Takesue N. (2023). All-Organic Polymer Dielectric Materials for Advanced Dielectric Capacitors: Theory, Property, Modified Design and Future Prospects. Polym. Rev..

[17] Adekunle A.A., Oparanti S.O., Fofana I. (2023). Performance Assessment of Cellulose Paper Impregnated in Nanofluid for Power Transformer Insulation Application: A Review. Energies.

[18] Khan S.A., Tariq M., Khan A.A., Alamri B. (2021). Effect of Iron/Titania-Based Nanomaterials on the Dielectric Properties of Mineral Oil, Natural and Synthetic Esters as Transformers Insulating Fluid. IEEE Access.

[19] Chan K.Y., Baktash A., Demir B., Mayes E.L., Yang D., Pham D.Q., Lin K.T., Mouritz A.P., Ang A.S., Fox B. (2021). Tailoring mechanical and electrical properties of graphene oxide film for structural dielectric capacitors. J. Power Sources.

[20] Tang R., Nomura K., Inoue K., Kotani M., Kyotani T., Nishihara H. (2022). Capacitance of edge-free three-dimensional graphene: New perspectives on the design of carbon structures for supercapacitor applications. Electrochim. Acta.

[21] Liu L., Qu J., Gu A., Wang B. (2020). Percolative polymer composites for dielectric capacitors: A brief history, materials, and multilayer interface design. J. Mater. Chem. A.

[22] Huang J., Sumpter B.G., Meunier V. (2008). A Universal Model for Nanoporous Carbon Supercapacitors Applicable to Diverse Pore Regimes, Carbon Materials, and Electrolytes. Chem. Eur. J..

[23] Yu Y., Li C., Wang H., Chen J., Zhu X., Ying Z., Song Y. (2022). High-specific-capacitance electrolytic capacitors based on anodic TiO2 nanotube arrays. Electrochim. Acta.

[24] Liu S., Wei L., Wang H. (2020). Review on reliability of supercapacitors in energy storage applications. Appl. Energy.

[25] Andrei O.E., Bica I. (2009). Some mechanisms concerning the electrical conductivity of magnetorheological suspensions in magnetic field. J. Ind. Eng. Chem..

[26] Bica I., Anitas E. (2018). Magnetic field intensity effect on electrical conductivity of magnetorheological biosuspensions based on honey, turmeric and carbonyl iron. J. Ind. Eng. Chem..

[27] Bica I., Anitas E. (2018). Magnetodielectric effects in membranes based on magnetorheological bio-suspensions. Mater. Des..

[28] Bica I., Anitas E.M., Chirigiu L. (2020). Hybrid Magnetorheological Composites for Electric and Magnetic Field Sensors and Transducers. Nanomaterials.

[29] Iacobescu G.E., Bunoiu M., Bica I., Sfirloaga P., Chirigiu L.M.E. (2023). A Cotton Fabric Composite with Light Mineral Oil and Magnetite Nanoparticles: Effects of a Magnetic Field and Uniform Compressions on Electrical Conductivity. Micromachines.

[30] Bica I., Anitas E.M., Choi H.J., Sfirloaga P. (2020). Microwave-assisted synthesis and characterization of iron oxide microfibers. J. Mater. Chem. C.

[31] Genç S. (2002). Synthesis and Properties of Magnetorheological (MR) Fluids. Ph.D. Thesis.

[32] Baabu P.R.S., Kumar H.K., Gumpu M.B., Babu K.J., Kulandaisamy A.J., Rayappan J.B.B. (2023). Iron Oxide Nanoparticles: A Review on the Province of Its Compounds, Properties and Biological Applications. Materials.

[33] Meijer J.M., Rossi L. (2021). Preparation, properties, and applications of magnetic hematite microparticles. Soft Matter.

[34] Malasi A., Taz H., Farah A., Patel M., Lawrie B., Pooser R., Baddorf A., Duscher G., Kalyanaraman R. (2015). Novel Iron-based ternary amorphous oxide semiconductor with very high transparency, electronic conductivity and mobility. Sci. Rep..

[35] Pal R. (2023). Recent Progress in the Viscosity Modeling of Concentrated Suspensions of Unimodal Hard Spheres. ChemEngineering.

[36] Batchelor G.K. (1977). The effect of Brownian motion on the bulk stress in a suspension of spherical particles. J. Fluid Mech..

[37] Felicia L.J., John R., Philip J. (2013). Rheological Properties of Magnetorheological Fluid with Silica Nanoparticles StabilizersA Comparison with Ferrofluid. J. Nanofluids.

[38] Chauhan V., Kumar A., Sham R. (2024). Magnetorheological fluids: A comprehensive review. Manuf. Rev..

[39] Bica I., Anitas E.M., Averis L.M.E., Kwon S.H., Choi H.J. (2019). Magnetostrictive and viscoelastic characteristics of polyurethane-based magnetorheological elastomer. J. Ind. Eng. Chem..

[40] Wu M., Xiong Y., Jia Y., Niu H., Qi H., Ye J., Chen Q. (2005). Magnetic field-assisted hydrothermal growth of chain-like nanostructure of magnetite. Chem. Phys. Lett..

[41] Zhang X., Lu W., Zhou G., Li Q. (2020). Understanding the Mechanical and Conductive Properties of Carbon Nanotube Fibers for Smart Electronics. Adv. Mater..

